# Exploring the vaginal ecosystem: insights into host-microbe interactions and microbial community dynamics

**DOI:** 10.1128/iai.00499-24

**Published:** 2025-08-11

**Authors:** Emily F. Landolt, Jéssica da Conceição Mendonça, Abbey E. Behler, Stephen W. Lumsdaine, Tamanna Jafar, Lindsey R. Burcham

**Affiliations:** 1 Department of Microbiology, University of Tennessee 189504, Knoxville, Tennessee, USA; 2 Department of Obstetrics and Gynecology, University of Tennessee Health Sciences Center College of Medicine 235699, Memphis, Tennessee, USA; University of California at Santa Cruz, Santa Cruz, California, USA

**Keywords:** vaginal microbiome

## Abstract

The vaginal tract is a complex environment that changes throughout various life stages. Recent advances have improved our understanding of the vaginal microbiota and the influence of host factors on microbial colonization. The vaginal niche is characterized by unique qualities such as high abundances of glycogen and mucin, low pH, active cellular immunity, and fluctuations in hormone signaling that support a complex microbiota. While traditionally thought to be dominated by *Lactobacillus* species, emerging research highlights a more diverse microbiota, including both commensal and potentially pathogenic microbes. Given the interconnectedness of the microbial and host factors in this environment, minor shifts can lead to significant downstream effects on health. This review takes an ecosystems approach to explore the multifaceted relationship between the vaginal mucosa, the microbiota, and influences of environmental factors on shaping the two. We discuss the contribution of hormone signaling in shaping microbial communities, concepts of vaginal community stability and dysbiosis, and the emerging understanding of microbial metabolism and cross-feeding dynamics within the vaginal tract. Additionally, we will examine the interactions between microbes and immune cells in the vaginal mucosa, including mechanisms by which the immune system modulates the local environment. By considering the feedback loops between the host and the resident microbiota, we propose key knowledge gaps and suggest interdisciplinary avenues for future research aimed at improving our understanding of vaginal health and disease. Understanding these complex interactions is important for advancing vaginal healthcare across all individuals.

## INTRODUCTION

The vaginal tract is a dynamic environment of critical importance for health and wellness throughout female adolescence, puberty, reproductive age (including non-pregnant, prenatal, and postpartum periods), and pre- and post-menopause. Though historically understudied, more recently, our knowledge of the vaginal microbiota and the physiological impact of the host on microbial colonization has drastically expanded (1). The vaginal ecosystem has unique identifying qualities including an abundance of glycogen and mucus as nutrient sources, a characteristic low pH, active cellular immunity, and hormonal signaling that fluctuates throughout puberty, menstruation, pregnancy, and menopause (2). The vaginal microbiota is largely thought to be dominated by beneficial lactobacilli, but more recent evidence has shown the vaginal lumen is home to a diverse array of microbes including commensal and potentially pathogenic bacteria, viruses, and fungi that vary in abundance. Importantly, these traits are strongly interconnected, and minor changes in this environment can lead to cascading downstream effects.

This review will utilize an ecosystem approach toward a more holistic understanding of the vaginal mucosa and the impact of environmental variables on microbial colonization and host-microbe interactions. Because of their interconnected nature, it is important to consider the impacts of the host and the microbes on each other within this niche. Discussion will highlight (i) the physiology of the system including the role of hormone signaling on nutrient availability and how changes can temporally affect microbial communities; (ii) emerging ideas of vaginal community stability and dysbiosis, including discussion of *Lactobacillus* dominance and vaginal pH in the context of colonization resistance, as well as recent metagenomic advancements toward understanding nutritional cross-feeding between microbes in the vaginal tract; and (iii) new perspectives on microbial interactions with immune cells in the vaginal mucosa and mechanisms by which the immune system modulates the vaginal environment. We will discuss feedback potential between the vaginal environment and the organisms that reside within and outline key gaps in knowledge, proposing integrated areas of investigation that will improve our understanding of vaginal health and disease.

## INTERFACE OF HORMONAL DYNAMICS, NUTRIENT AVAILABILITY, AND THE MICROBIOTA

Reproductive hormone signaling is known to drive physiological and environmental change that play critical roles in shaping the vaginal microbiome (3). The menstrual cycle is divided into follicular, which contains menstruation and proliferation, ovulatory, and luteal phases that are marked by fluctuations of estrogen, progesterone, luteinizing hormone, and follicle-stimulating hormone that regulate the thickening of the uterine lining and the release of eggs from the ovaries for fertilization (4). The vaginal environment is sensitive to the natural hormonal variation that occurs throughout the menstrual cycle and across the gynecological timeline, and directional change of individual hormones can affect resistance or susceptibility to genitourinary infection (5–8). The interface between microbes and host hormone production is the basis of an emerging field called microbial endocrinology, which seeks to understand the impact of host hormones on bacterial growth and virulence (9–12). This section will describe temporal dynamics within the vaginal mucosa in the context of hormone production and discuss their impact on factors such as nutrient availability, barrier integrity, and microbial community structure.

### Menstruation impacts nutrient availability and vaginal community stability

**Fig 1:**
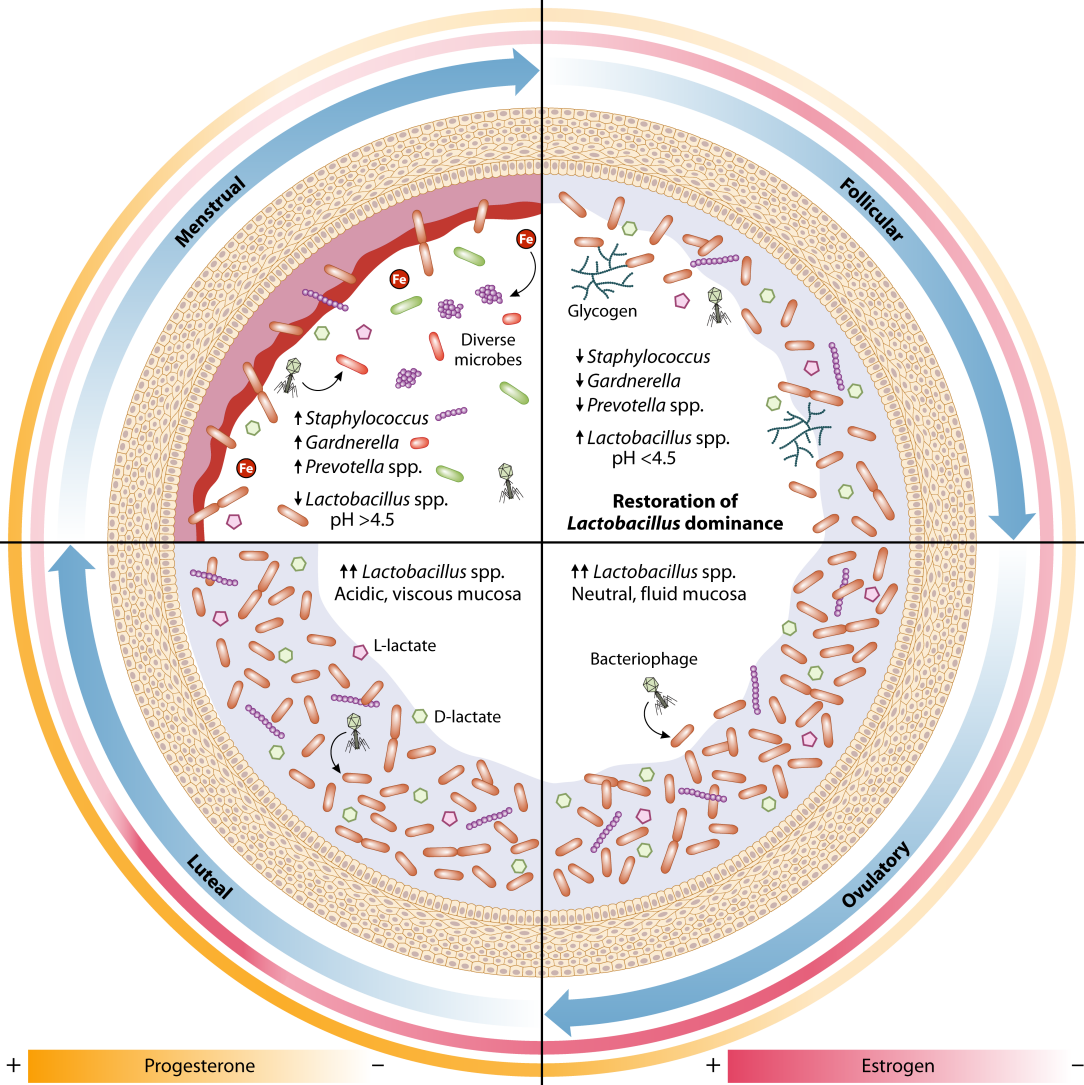
Hormone signaling during the menstrual cycle affects environmental and microbial dynamics. Decreased estrogen and blood flow during menstruation contribute to increased microbial diversity in some individuals. The increase in estrogen during the follicular phase leads to glycogen deposition that supports the growth of *Lactobacillus*. During ovulation, the mucosa experiences increased hydration, which reduces viscosity and leads to a more neutral pH. The progesterone surge during the luteal phase is marked by a more rigid, less-hydrated mucosal barrier that can function to trap pathogens, also creating an abundant nutrient source for vaginal microbes.

The follicular phase of the menstrual cycle is composed of two key parts: menstruation and proliferation (4, 13). During menstruation, microbial communities can be significantly altered, leading to increases in bacterial diversity and richness (Fig. 1) (6, 14). Although the physiological alterations during menstruation can lead to community changes, they vary considerably across individuals, which led to the establishment of four vaginal community dynamics (VCDs) that describe temporal changes in the vaginal microbiota in the context of the menstrual cycle (15). VCDs were established for microbial profiles of “constant eubiosis” or “constant dysbiosis,” with more than 80% of daily samples dominated by *Lactobacillus crispatus* or *Lactobacillus jensenii*, or dominance by *Lactobacillus iners* or communities with high diversity, respectively. Individuals with samples below the 80% threshold of eubiosis or dysbiosis were binned into a subgroup of “unstable” vaginal community (15). Interestingly, 12% of individuals made up a distinct subgroup of “menses-related dysbiosis”, defined as having >80% of eubiotic samples during the proliferative, ovulatory, and luteal stages of the menstrual cycle and detectable dysbiosis during menstruation (15). Additional studies observed changes in community structure, with decreases in lactobacilli and increased diversity during menses (6, 16). A longitudinal cohort analysis from 32 reproductive-age individuals over 16 weeks described the lowest levels of microbial community constancy as menses-related (16), and some individuals experienced menses-related shifts from *L. crispatus*-dominant to microbiomes dominated by *L. iners* or *Streptococcus* (16). These changes in community structure during menstruation are likely due to a decrease in luminal glycogen deposition and due to the influx of menstrual blood products, whose impact on the vaginal microbial communities is proposed to be twofold. First, the influx of heme-bound iron during blood flow could potentially promote a shift from *L. crispatus* dominance to dominance by *L. iners*, whose growth may be supported by iron ions (17, 18). The second environmental change associated with menstrual blood flow is a physiological change in pH. In addition to the increased abundance of iron, the influx of more alkaline blood results in a neutral vaginal pH of 7.2–7.4 (7, 19). Together, these changes have been associated with transient colonization of opportunistic pathogens such as *Staphylococcus aureus* and *Streptococcus* spp., and the establishment of BV-associated bacteria like *Gardnerella vaginalis*, *Prevotella* spp., *Fannyhessea vaginae*, *Sneathia amnii*, *Ureaplasma parvum*, *Veillonella montpellierensis*, and *Peptostreptococcus* spp. (7, 14, 15, 19–22). It has not been clearly demonstrated that the association between menstruation and microbial diversity is directly dependent on one singular effect like an increase in nutrient iron, glycogen availability, or pH. These changes could be downstream effects of hormone fluctuation, or could be due to a physical disruption of the community as a result of shear force from blood flow—though it seems most likely that they collectively influence community outcomes.

### Proliferative phase: impacts on the epithelial barrier integrity and glycogen availability

The increased abundance of *Prevotella* and *Gardnerella* observed during the menstrual phase persists into the beginning of the proliferative phase, where estrogen levels are still relatively low (23); however, toward the end of the follicular phase and during the preovulatory stage, a restoration in *Lactobacillus* dominance has been observed that coincides with a peak in estrogen levels prior to ovulation (Fig. 1) (4). The impacts of estrogen production extend outside the menstrual cycle, and at the start of puberty, the production of estrogen increases, leading to the maturation and proliferation of the vaginal epithelium (24). The thickness of the vaginal epithelium peaks during the reproductive years and forms a multilayered barrier of loosely connected cells rich in glycogen (25, 26). Cells at the apical surface are regularly lysed and exfoliated, which results in the release of stored glycogen into the vaginal environment (13, 27). The recovery of *Lactobacillus* dominance that occurs in the late follicular phase is thought to correlate with the increased availability of luminal glycogen and remains stable throughout the luteal phase (14–16, 23).

### Vaginal glycogen availability and host and microbial degradation

Glycogen is a complex molecule consisting of α−1,4-glycosidic bonds and α−1,6-glycosidic branches, and concentrations in the vaginal tract fluctuate throughout the menstrual cycle and vary broadly across individuals. The unique abundance of glycogen within the vaginal lumen has intrigued researchers since the early 1900s, and it was proposed in 1908 that vaginal lactobacilli may be capable of utilizing glycogen as a carbohydrate source (28, 29). However, due to its large complex structure, microbes are unable to mobilize glycogen across their membranes, rendering it essentially useless in its raw form (30). Extracellular enzymatic activity is necessary for the breakdown of glycogen molecules into simpler sugars like dextrins, maltose, and maltotriose, creating reservoirs of “common goods” that become available for use by members of the microbiota (30–32). Vaginal fluids have detectable levels of host- and microbially derived glycogen degrading enzymes including α-amylases and pullulanases capable of cleaving α−1,4 glycosidic and α−1,6 glycosidic bonds, respectively. Host-derived α-amylases are found abundantly in the vaginal lumen and the endocervix (33–35).

Where microbial glycogen utilization was originally thought to be associated primarily with protective *Lactobacillus* spp. and several studies have reported positive correlations between glycogen levels, the abundance of *Lactobacillus*, and low vaginal pH (24, 31, 33, 36), not all *Lactobacillus* isolates have been shown to use glycogen (37–39). More recent studies have identified glycogen-degrading enzymes encoded across bacterial genomes from samples representative of all vaginal community state types (CSTs) (38, 40–43), and glycogen is now known to serve as an energy source for both beneficial and pathogenic microorganisms during colonization or infection of the vaginal lumen (31, 32, 43). One study identified higher concentrations of α-amylase in the vagina of individuals with high *Lactobacillus* abundance, and inversely, lower detectable concentrations of α-amylase in individuals with decreased *Lactobacillus* abundance or bacterial vaginosis (BV)—a medical diagnosis marked by a decrease in *Lactobacillus* abundance and an increase in the abundance of anaerobic bacteria (33). This suggests that α-amylase production could potentially support *Lactobacillus*-dominant communities. In contrast, another study observed a 5.4-fold increase of α-amylase in vaginal secretions of first trimester pregnancies when the vaginal microbiota was dominated by *L. iners* rather than *L. crispatus* (44). It was proposed that increased production of α-amylase could be an effort by the host to restore *L. crispatus* dominance and stabilize the microbial community (44).

Other commensal or pathogenic organisms found in the vaginal tract including *Gardnerella* spp. (43, 45, 46), *Streptococcus agalactiae* (47, 48), *Candida albicans* (49), and *Trichomonas vaginalis* (50–52) have also been shown to express glycogen-degrading enzymes suggesting that breakdown of glycogen into usable sugars might be an important physiological process for community members besides the lactobacilli. It is noteworthy that some organisms express glycogen-degrading enzymes that exhibit optimal activity at lower pH, which would hypothetically be active in a *Lactobacillus*-dominant environment, whereas other organisms produce amylases which function optimally at a more neutral pH range of 6–7 (32, 53). This suggests that diverse vaginal microbes may be able to utilize glycogen across fluctuating conditions of the lumen, like what they may experience during different phases of the menstrual cycle or in periods of microbial dysbiosis. Community production of secreted glycogen-degrading enzymes also presents an opportunity for nutrient cross-feeding, where community members that are not capable of catabolizing glycogen on their own can benefit from active transport of breakdown products, as described in *Enterococcus faecalis* (54).

Though it is evident that glycogen availability has an important role in shaping community structure and function in the vaginal microbiota, there are key gaps in knowledge in understanding how this occurs. Understanding past association, how glycogen availability impacts community dynamics and turnover, and probing the directionality of glycogen production—is glycogen supporting *Lactobacillus* growth or is *Lactobacillus* dominance promoting formation of a healthy epithelial barrier with rich glycogen stores—will be important for appreciating the full impact of glycogen metabolism on vaginal health.

### Ovulation and the luteal phase: impacts on the vaginal mucosal barrier

Ovulation occurs directly after the estrogen surge of the proliferative phase and signals the start of the luteal phase, marked by a parallel rise in progesterone and a low secondary peak in estrogen (4). An important environmental variable regulated by hormone signaling during the ovulatory and luteal phases is the physical properties of the vaginal mucosal barrier (55). The mucosal barrier is formed by dense hydrogel networks of mucin glycoproteins decorated with inorganic ions, defensins, and immunoglobulins called the cervicovaginal mucosa (CVM) (56). Mucin glycoproteins are produced in two forms, the cell-surface anchored and the secreted, gel-forming and are heavily O-glycosylated, creating a large, negatively charged physical barrier (57). Vaginal and ectocervical epithelial cells produce surface-anchored MUC1 and MUC16, while the endocervical epithelium and cervical goblet cells are primarily responsible for the secretion of gel-forming mucins, MUC5B, MUC5AC, and MUC6 (58–61). Another cell-anchored mucin, MUC4, was found to be highly expressed in the endocervix (60), while a more recent proteomics study detected anchored mucins, MUC1 and MUC16, but failed to detect peptides of MUC4 from cervical mucus secretions (62), making its impact in the CVM unclear. However, it is important to note that the detection of mucins by proteomics can be challenging, and discrepancies across studies could be due to technical challenges rather than biological absence.

Production of mucus, specifically MUC5B mucins, is highest at ovulation and well-hydrated (59, 62), resulting in the formation of estrogenic mucus that is thin and watery and has a more neutral pH, facilitating passage of sperm for fertilization (62, 63). When progesterone peaks during the luteal phase, production of mucus decreases and continues to decline throughout the later portion of the menstrual cycle (19). In the luteal phase, mucus loses fluidity leading to the “progesterone effect,” or loss of mucosal spinnbarkeit (elasticity) (55), resulting in a thick, viscous, negatively charged barrier that functions primarily to protect the underlying epithelium and the upper reproductive tract from pathogen invasion (56, 64–68). Studies have shown that CVM contains IgG and inhibits movement of human immunodeficiency virus type I (HIV-1), serving in a protective role against viral transmission (67, 69, 70). Interestingly, mucosal communities dominant for *L. crispatus* were more effective at reducing viral motility than communities dominated by *L. iners* or dysbiotic communities (67). Another study reported that mucosal surfaces with bacteriophage present serve as an additional protective barrier against pathogen invasion, specifically *Escherichia coli*, proposing the phage-mucosal interface as an example of non-host-derived immunity (71). During pregnancy, mucins, primarily MUC5B and MUC5AC, form the cervicovaginal mucus plug that serves as a physical barrier separating the microbe-rich vaginal lumen from the uterine space and has antimicrobial peptides like lactoferrin, lysozyme, and calprotectin; immunoglobulins; and viscoelastic properties that function to protect against pathogen ascension (72–76). Loss of Muc5B in murine models resulted in increased bacterial ascension to the uterus, decreased mucosal barrier integrity, and an increased rate of preterm birth during infection (75, 77).

In addition to serving as a barrier to infection, mucins can serve as a substrate for attachment and as a nutrient source for a diverse range of microbes in the vaginal lumen (71, 78–81). *S. agalactiae* and *E. faecalis* express pilus structures that bind mucins directly and promote persistence in the vaginal and gastrointestinal tracts (77, 82, 83). Similarly, many species of *Staphylococcus* and *Lactobacillus* are capable of binding mucins, and a unique mucin-binding domain has been described exclusive to lactic acid bacteria (LAB) that is predicted to be involved in mucin attachment or degradation (78, 80, 84, 85). Expression of mucins and goblet cell secretion is known to increase following infection as a type of innate immune defense to secure the mucosal barrier and is dependent on nuclear factor kappa B ( NFкB) signaling (86, 87). But microbes have evolved in the presence of mucins and express many glycosidases capable of degrading mucin glycans for use as a nutrient source (81, 88–93). In the context of the vaginal mucosa, it was thought that production of enzymes such as sialidases was exclusive to BV-associated pathogens, due to the negative effect they have on degradation of the mucosal barrier, which reduces viscosity, making the environment more susceptible to infection (94–97).

Studies have consistently detected increased glycosidase and sialidase enzymatic activity in women with BV (87, 88, 96), and metatranscriptomic analyses observed the highest levels of sialidase expression in samples from individuals with a vaginal microbiota clustering to CST IV (98). Increased sialidase activity associated with BV or vaginal dysbiosis is associated with depletion of N- and O-linked glycans and has negative impacts on mucosal barrier integrity and can exacerbate inflammation (68, 99, 100). Sialidase enzymes were thought to be produced primarily by *Gardnerella* and *Prevotella* spp. during BV (98, 101), but a recent study detected transcripts for bacterial sialidases at high percentages in samples representative of all vaginal CSTs (98), suggesting expression of sialidases may have a role in community physiology aside from pathogen invasion. To support this, there are examples of sialidase activity promoting growth and nutrient cross-feeding in communities, as is the case with *Fusobacterium nucleatum* sialidase production contributing to vaginal colonization and promoting growth of *G. vaginalis* in the vaginal tract (79). Cross-feeding of mucin breakdown products has been characterized more extensively in the context of the gastrointestinal tract. *Akkermansia muciniphila*, as the name implies, is known for its preference for mucus as a nutrient source and has been shown to produce several sialidases and fucosidases that promote cross-feeding with butyrate-producing *Clostridia* (81). Similarly, sialidases produced by *Bifidobacterium* spp. encourage mucin cross-feeding with other gastrointestinal *Bifidobacterium* (102). In the context of the vaginal tract, co-colonization with *A. muciniphila* promotes *S. agalactiae* persistence, and metabolic modeling revealed a potential for nutrient exchange between the two organisms, although it remains unclear if this is due to immune modulation, communication between the species, or metabolic cross-feeding (103).

### Postpartum and menopausal hormone changes impact microbial community structure

The postpartum and postmenopausal stages are important physiological phases but are understudied areas of reproductive health. The postpartum interval is marked within days of delivery by a sharp decline in estrogen concentrations, with common symptoms including bleeding, uterine contractions, and fatigue (104–106). Menopause is the slowing and eventual stop of the menstrual cycle, typically begins after age 45, and is characterized by a decrease in estrogen production, irregular menstrual cycles, vaginal dryness, and a heightened susceptibility to irritation and infections (13, 107–110). During the postpartum and post-menopausal periods, the decline in estrogen levels often results in a decrease in the dominance of lactobacilli (24, 26, 107, 109, 111, 112). The vaginal microbiota during the postpartum interval often transitions toward diverse states, with notable enrichment of *L. iners*, *F. vaginae*, *G. vaginalis*, *Finegoldia magna*, and *Prevotella* spp. (104, 111, 113–115), which in some individual microbiomes remained highly diverse more than 1 year after delivery (104). Similarly, postmenopausal individuals experience decreases in estrogen and progesterone and can have lowered abundance of lactobacilli and significant increases in the abundance of intermediate BV-associated species, including *S. agalactiae*, *Staphylococcus epidermidis*, and *Corynebacterium pyruviciproducens* (111, 116). Hormone replacement therapy is an available treatment option for the postmenopausal population and is thought to stimulate epithelial cell maturation, increase carbohydrate availability to support the native microbiota, and decrease microbial diversity (112, 117–120). However, there is still a significant need to expand our understanding of vaginal health in these populations.

## VAGINAL COMMUNITY STABILITY, DYSBIOSIS, AND RESILIENCE

### Evolving perspectives on VCDs

Vaginal CSTs were first described from 16S rRNA sequencing of a North American cohort of reproductive age women across four ethnic backgrounds (40). Clustering of the microbial communities identified five distinct groups designated CST IV. CST I was dominated by *L. crispatus*, whereas groups II, III, and V were dominated by *Lactobacillus gasseri*, *L. iners*, and *L. jensenii*, respectively. *Lactobacillus* dominance is often described as protective, with the exception of the *L. iners*-dominant CST III, which may be considered a transitional state suggesting the microbial community dynamics are in flux between an optimal, suboptimal, or BV state (121, 122). CST IV represented more diverse microbial communities, with an increased representation of anaerobic bacteria from genera *Prevotella, Dialister, Fannyhessea*, *Gardnerella*, *Megasphaera*, *Peptoniphilus*, *Sneathia*, *Eggerthella*, *Aerococcus*, *Finegoldia*, and *Mobiluncus* (40). With the advent of new technologies and new perspectives, the classifications of CSTs have expanded. Development of a tool called VALENCIA categorized samples based on their similarities and stratified the original five CSTs into expanded CSTs that provide varying levels of resolution, allowing for inclusion of state types representative of high and low abundances of focal species and incorporation of some details of complex mixed communities (116). Another study introduced a mixed membership topic model to study population structure, consider longitudinal changes of the microbiome, understand the impact of pregnancy, and identify subcommunities. Using a large cohort of vaginal swabs collected from pregnant and non-pregnant populations, nine subcommunities were identified. Four were marked by dominance of *Lactobacillus* spp. (*L. crispatus*, *L. jensenii*, *L. iners*, and *L. gasseri*), and the remaining five were non-*Lactobacillus* subcommunities with diverse presence of *Streptococcus*, *Prevotella* spp., *Gardnerella* spp., *Corynebacterium* spp., *F. vaginae*, *Finegoldia*, etc. (123).

Recent advances in metagenomics and metatranscriptomics are increasing functional analyses of communities at the species level, allowing for more resolution of the spatiotemporal dynamics of the vaginal microbiota (124). Recent introduction of 27 distinct metagenomic CSTs (mgCSTs) provided critical information on potential microbial community function and has moved the field past the question of ‘‘what microbes are there” to “what are they doing” (125). Although the CST approach provides important insights into the vaginal microbiota composition at specific moments in time, the simplification of the vaginal microenvironment as static rather than dynamic is somewhat of a barrier to better understanding and acknowledging the diversity of the microbial population. As previously mentioned, the establishment of VCDs was suggested to address temporal changes, based off of the CST system but incorporating longitudinal sampling to cluster menstrual phase with bacterial species and co-occurring bacteriophages of the vaginal microbiome (15). The general definition of “constant eubiosis” was associated with >80% detection of the CSTs I and V, while “constant dysbiotic” individuals were associated with >80% detection of CSTs III and IV (15). The categorization of VCDs brought important new perspectives on vaginal microbial dynamics, identifying *E. coli* as present in a twofold higher abundance in the eubiotic communities compared to the unstable or menses-dysbiotic clusters and identifying *S. agalactiae*, *L. iners*, and *U. parvum* as higher abundance in the temporally-sensitive unstable and menses-dysbiotic states. The inclusion of bacteriophage identification showed “eubiotic” individuals had a 10-fold increase in phage abundance compared to the other VCDs, though this population may be low diversity (126). These studies continue to advance our understanding of the vaginal microbiome and reinforce the inherent microbial diversity that exists in this environment.

### Colonization resistance: a protective role for lactobacilli

The characteristic low pH of the vaginal tract is attributed to the ability of both host and vaginal microbes to ferment glucose and glycogen into lactic acid resulting in acidification of the vaginal environment (19, 127, 128). The vaginal epithelium also serves as a source of lactic acid production through the production of L-lactate, but D-lactate has been shown to make up the majority of vaginal lactate (129–131). Lactic acid is the primary metabolic end product derived from sugar fermentation by LAB including three of the most common lactobacilli, *L. crispatus*, *L. gasseri*, and *L. jensenii,* which produce high levels of D-lactate, while sequenced *L. iners* genomes lack a D-lactate dehydrogenase (128, 132, 133). Acidification of the vagina by microbially derived lactic acid is an example of colonization resistance (134), whereby lactobacilli and their metabolic end products decrease the environmental pH and promote barrier integrity, fostering a protective environment that supports growth of *Lactobacillus* while inhibiting the proliferation of other bacterial species (3, 53, 68, 135, 136). However, one study has shown that lactic acid production has no protective antimicrobial properties against pathogenic anaerobes when the pH is increased, suggesting that both the production of lactic acid and the acidification of the environment are necessary for successful colonization resistance (137, 138).

Change in vaginal pH is considered one of the primary indicators of bacterial dysbiosis, and increased pH (>4.5) is commonly associated with the presence of anaerobes such as *Gardnerella*, *Prevotella*, and *Sneathia* (139). The presence of BV-associated bacteria that are capable of degrading and metabolizing the negatively charged mucosal barrier can impact the thickness of the mucus layer, leading to an increase in vaginal pH (140). The menstrual cycle can also impact vaginal pH. As mentioned above, it is intricately linked to glycogen availability, mucosal integrity, and alkaline blood products, temporarily resulting in an increase in vaginal pH that can allow for the outgrowth of potentially pathogenic organisms (7, 19, 140). Although it has been widely accepted that the vaginal pH of patients with *Lactobacillus*-dominant communities is commonly <4.5 (141), recent findings suggest that a large percentage of individuals with *Lactobacillus* mgCSTs can present with decreased acidification. Specifically, the metagenomic subspecies of *L. crispatus* (mgSs2) was associated with individuals with vaginal pH above 4.5, thought to be due to the loss of a second D-lactate dehydrogenase enzyme (125). Further, this robust study including 1,890 vaginal samples described 16/27 mgCSTs as *Lactobacillus*-dominant; however, a surprisingly low percentage, 31%, of samples were considered to be a low vaginal pH of <4.5 and 69% of samples with pH >4.5, which is a clinical indicator of vaginal dysbiosis or BV (125). Some *Lactobacillus* species produce bacteriocins and hydrogen peroxide, which have been proposed to contribute to colonization resistance (142–144); however, recent evidence has suggested that hydrogen peroxide levels in the vaginal tract are likely not high enough to play a critical role in the inhibition of pathogenic microbes, and the majority of the colonization resistance attributed to *Lactobacillus* is due to pH or other uncharacterized factors (145).

### States of vaginal dysbiosis: BV, AV, VVC, and CV

BV is the most common type of vaginitis experienced among reproductive age women (141, 146), with a worldwide prevalence of 23%–29% of women affected (146–148). BV is associated with a low abundance of *Lactobacillus* spp. and an overgrowth of anaerobic bacteria from genera such as *Gardnerella*, *Prevotella*, *Sneathia*, *Mobiluncus*, *Fannyhessea*, and *Finegoldia* to name a few (22, 149). Individuals who present with BV-associated microbes are at an increased risk of acquiring sexually transmitted infections (STIs) and HIV, adverse pregnancy outcomes, and cervical dysplasia (141, 150, 151), and while screening for BV is not recommended during pregnancy, a meta-analysis study indicated that BV-positive individuals have twice the risk of preterm labor and nine times higher risk of spontaneous abortion (152, 153). BV is diagnosed by Amsel’s criteria (154, 155) or Nugent score (156, 157). Amsel’s criteria assess clinical signs and symptoms including the presence of thin homogeneous discharge, vaginal fluid pH >4.5, discharge with a fishy odor (i.e., the whiff test), and presence of “clue cells” by examination of vaginal epithelial cells with adherent bacteria (154). Nugent scores are based on Gram stain interpretation of microbial morphology from a vaginal smear (156). In addition to these standard methods, multiple point-of-care and molecular-based tests are commercially available; however, they are still recommended for use in association with a standard method of diagnosis (141). Although these methods can be useful at identifying patients with active BV, there are disparities between the diagnosis and the symptoms experienced. A recent metagenomic study found that of patients who were BV-positive based on Amsel’s criteria, only 12.3% had confirmed BV and were actively symptomatic (125).

BV is marked by a decrease in luminal lactic acid and an increase in the abundance of short-chain fatty acids (SCFAs) that are generally produced by anaerobic members of the microflora through fermentation and amino acid catabolism (158–162). SCFAs in the gut have been shown to have anti-inflammatory effects; however, recent work has demonstrated that SCFA exposure, representative of BV-associated metabolites, induces production of proinflammatory cytokines in the vaginal epithelium and reduces epithelial barrier integrity (136, 163). It has also been shown that SCFAs in the gut increase induction of prophage elements in *Lactobacillus reuteri* (164). Long chain fatty acids (LCFAs) are common in mammalian mucosal surfaces and have been detected in swab samples collected from the vaginal lumen (165). Studies performed *in vitro* have demonstrated that LCFAs, specifically oleic acid, can inhibit growth of *L. iners* and other BV-associated bacteria and may promote growth of *L. crispatus* and other beneficial lactobacilli (166). These data suggest that both long and SCFAs and fatty acid metabolism may have important roles as potential drivers of vaginal community structure.

In contrast to BV, the vaginal community can also become dominated by aerobic, opportunistic pathogens that promote the increase of pro-inflammatory markers such as interleukin (IL)-6, a state that has been termed aerobic vaginitis (AV) (167, 168). The prevalence of AV is lower than BV, ranging between 7% and 12% of the population (169). Similar to a BV diagnosis using Nugent scores, a scoring system is used to diagnose AV, with wet mount phase contrast microscopy (170). The diagnostic criteria are based on the presence of lactobacilli, the quantification of inflammatory cells, and the morphology of the epithelium (170). The species that are most commonly associated with AV include *E. coli*, *S. agalactiae*, *E. faecalis*, *Klebsiella pneumoniae*, coagulase-negative *Staphylococcus* (such as *S. epidermidis*), and *S. aureus* (169, 171).

*Candida* spp., primarily *C. albicans*, are the most common fungal members of the vaginal microflora and, like many bacterial species, can persist as commensals at basal levels. Overgrowth of *Candida* can arise, resulting in vulvovaginal candidiasis (VVC), which is marked by vaginal soreness or discomfort, vaginal itching, and abnormal vaginal discharge (141). Like any member of the vaginal microbiota, community dynamics and environmental factors influence the growth of *Candida* spp., and interkingdom interactions are often driven by the presence of *Lactobacillus* spp. and host elements (172), for example, biofilms formed by *Lactobacillus* can maintain the commensal yeast form of *Candida* rather than the virulent, hyphal form (173). While the effects of VVC and the impacts of *Candida* on the immune system have been well characterized, there is still much to uncover about the role of commensal fungi in the vaginal microbiome. Cytolytic vaginosis (CV), which is caused by an overgrowth of *Lactobacillus* and an increase of lactic acid above normal, is primarily caused by *L. crispatus*. CV presents with similar symptoms and is often misdiagnosed as VVC (174–176), leading to improper treatment that often results in persistent symptoms. CV is an interesting pathology that suggests community balance is a more important factor for eubiosis rather than purely *Lactobacillus* dominance.

The recommended treatment for BV and AV is prescription antibiotics for symptomatic patients (141, 177). Although this is often the first choice of treatment, the indiscriminate use of antibiotics affects pathogenic and beneficial bacteria, disrupting the natural balance of bacteria in the vagina and increasing the susceptibility to secondary infections including VVC (141, 178). Alternative options have been proposed for the treatment of BV, including probiotic use of *Lactobacillus rhamnosus* or *L. crispatus* strains (179, 180) and recombinant, phage-derived endolysins that could selectively target BV-associated microbes like *G. vaginalis* (181, 182). Conflicting opinions still exist in the field on whether BV, AV, and CV should be considered infectious diseases and are normal fluctuations of bacterial community states or syndromes (183). Some of the discussion points raised suggest more consideration be placed on patients with clinical BV diagnosis by Amsel’s criteria or Nugent scores who present with variable or inconsistent symptoms, or asymptomatically even with detectable bacterial species like *G. vaginalis*. As the field advances, these discussions should continue, especially in cases where antibiotic interventions are introduced for asymptomatic individuals with “dysbiotic or non-optimal” microbial communities.

### Understudied microbes in the vaginal microenvironment

Our current understanding of microbial communities in the vaginal tract is primarily defined by dominant species. Oftentimes in large data sets, species of rare abundance are poorly represented or are grouped into non-informative categories such as “other.” The idea of the ecological concept of keystone species is an understudied and emerging area of investigation in the vaginal microbiome field. This concept suggests that species of low abundance may have effects on microbial communities that are disproportionate to their abundance (184–186). More recent efforts have sought to acknowledge the importance of these low-abundance species to the vaginal ecosystem (15, 185, 186). Examples include *A. muciniphila*, which promotes persistence of vaginal colonization by *S. agalactiae* and may potentially influence birth outcomes; Atopobiaceae family and *Fannyhessea vaginalis* are commonly found in patients with cervical cancer and correlated with increased cancer immune biomarkers; *Sneathia* spp. are associated with higher rates of cervical cancer and HPV in Hispanic individuals; *Mobiluncus mulieris*, whose extracellular vesicles stimulate pro-inflammatory cytokine production in vaginal and cervical cells; and *F. magna*, which has been shown to activate neutrophils and trigger the release of neutrophil extracellular traps (NETs), among others (187–191). In addition to the low-abundance bacterial species found in the vaginal tract, outside of STIs, viruses that inhabit this environment are often overlooked, though they likely play an important role in the vagina (192).

Studies assessing the vaginal virome have noted only 4%–6% of total reads belonged to eukaryotic viruses, and the remaining 94%–96% aligned to bacteriophage (126, 193). These clinical studies have revealed associations between the bacteria present and their respective bacteriophage in the vaginal tract, showing that bacteriophage community composition is a strong predictor of BV and associating the presence of specific phages, like *Bacillus* viruses *Camphawk* and *Pony,* with a BV diagnosis (126). Phage taxonomy follows closely with the host bacteria present in the environment, and phages have been shown to cluster into a low phage diversity, associated with *Lactobacillus-*dominant community, and a high phage diversity, associated with non-*Lactobacillus* dominance (15, 126). Another study showed phages in the vaginal tract of mid-gestation pregnancies were found to be of *Lactobacillus*, *Streptococcus*, *Staphylococcus*, and *E. coli* origin rather than BV-associated microbes, but no link existed between specific phage families and CSTs (194). Although there are some clear associations between the bacteriophage and bacterial communities present in the vaginal tract, it remains unclear if bacteriophages are involved in driving bacterial community changes or if the presence of specific bacteriophage is an artifact of changes having already occurred. Future studies including longitudinal sampling of the vaginal microbiome can begin to elucidate the dynamics between the viral and bacterial communities.

## MICROBIAL INTERACTIONS AND ENVIRONMENTAL CHANGES ASSOCIATED WITH THE IMMUNE RESPONSE

### Vaginal microbiota and immune cell interactions in the vaginal tract

**Fig 2:**
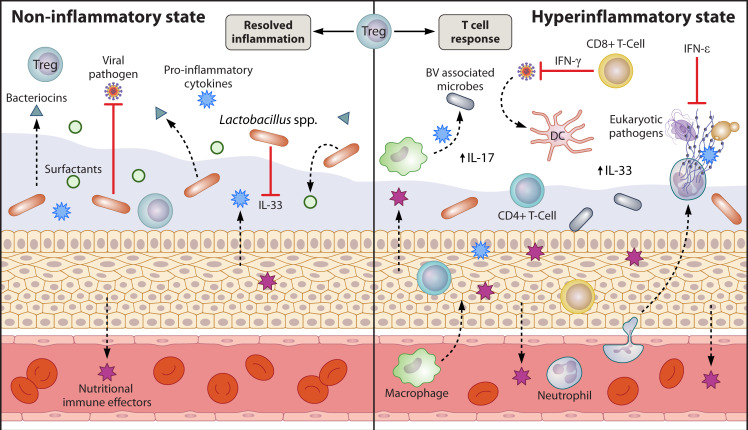
In conditions of increased inflammation, in response to bacterial, viral, or eukaryotic pathogens or immune dysregulation, the host epithelium and immune cells secrete pro-inflammatory cytokines or nutritional immune effector molecules. NETosis contributes to pathogen clearing, and antigen presentation by DCs facilitates production of IFN-γ by CD4^+^ and CD8^+^ T cells, contributing to effective immune activation in response to viral infection, and increased IFN-ε protects against bacterial, viral, and eukaryotic pathogens. Increased IL-17 is detected in environments of microbial diversity or infection, though the specific source is not yet known. In environments with decreased inflammation, there is an increase in surfactant production and a dampened concentration of CAMPs and nutritional effector molecules, and lactobacilli suppress production of the alarmin IL-33 that protects against viral infection. Induction of IL-33 expression and IFN-γ production, rendering the host more susceptible to viral invasion. Tregs help to regulate immune homeostasis; they are critical for mounting an effective immune response and are involved in the resolution of inflammation to protect from immune-mediated damage.

The vaginal tract has a robust cellular immune repertoire that significantly impacts the environment (Fig. 2), including CD8^+^ T cells and CD4^+^ T cells that account for up to 50% of leukocytes in the vaginal tract (195, 196). Reproductive tract T cells have primarily been studied in the context of STIs. The CD8^+^ tissue resident memory lymphocytes monitor for pathogen invasion and, upon recognition and activation, release interferon gamma (IFN-γ), initiating recruitment of immune cells (197–200). Regulatory T cells (Tregs) are a critical cellular player involved in mounting an appropriate immune response and facilitating successful recruitment of T cells, natural killer cells, and dendritic cells (DCs) during active infection (201–203). Tregs have an important role in monitoring the inflammatory environment of the reproductive tract, and upon activation, Tregs can suppress inflammatory cytokine production, produce anti-inflammatory IL-10, and produce granzyme B, a serine protease that helps to control immune-associated tissue damage and promotes resolution of inflammation (201, 203–205). HIV-positive individuals have decreased abundance of Tregs and display expansion of inflammatory mucosal (Tim) CD8^+^ T cells that contribute to heightened and persistent inflammation in the vaginal environment (206). This increased T cell-mediated inflammatory environment was also observed in neovaginas of transwomen having undergone vaginoplasty, compared to *cis* vaginas (207). Changes in T-cell function and increased expression of proinflammatory markers were observed in Th17 cells from BV-positive individuals compared to BV-negative individuals (208). These chronic states of inflammation can increase susceptibility to secondary infections like STIs and BV and acquisition of HIV (206, 209).

Other leukocytes, including neutrophils, monocytes, macrophages, and DCs, are present and function together to regulate vaginal immunity (196, 210). During pathogen invasion in the vaginal tract, neutrophils are often the first immune cells recruited and have several mechanisms to kill pathogens, including degranulation, phagocytosis, and formation of NETs. NETs can ensnare pathogens, and NETosis, the formation of NETs, occurs in response to viral, fungal, and parasitic vaginal infections (211–214). Cervicovaginal swabs and cervicovaginal lavage fluid collected from individuals with non-*Lactobacillus*-dominant vaginal microbiota display increased abundance of proinflammatory cytokines and neutrophil-associated markers and decreased expression of occludin and desmoglein-1 which reflect decreased epithelial barrier integrity (208, 215–217). Similar phenotypes of increased proinflammatory toll-like receptor-mediated signaling and decreased pathways regulating barrier integrity were observed in neovaginas of transwomen compared to *cis* vaginas (218). Direct experiments assessing the functional properties of the vaginal epithelium in mice observed increased neutrophil recruitment and activation in mice challenged with BV-associated microbes, *M. mulieris* and *G. vaginalis* (215).

Antigen-presenting cells (APC) like macrophages, DCs, and Langerhans cells (LCs) also play an important role in regulation of vaginal immunity in response to pathogens (195, 196, 219). Vaginal microbial communities influence macrophage polarization, with *Peptostreptococcus anaerobius* promoting an anti-inflammatory M2 polarization (220), while in pregnant mice, *C. albicans* drives a pro-inflammatory M1 polarization of placental macrophages thought to contribute to the risk of adverse pregnancy outcomes associated with candidiasis (221). Maturation of DCs occurs as a result of activation through pattern recognition receptor signaling, and DC activation is increased following exposure to BV-associated bacterial species or cervicovaginal lavage collected from individuals with BV compared to lavage from healthy individuals (222, 223). DC activation does not occur broadly in response to all vaginal microbes because exposure to *L. crispatus* fails to induce activation (223). DCs are also important for the response to viral infection, and submucosal DCs, but not LCs, are responsible for antigen presentation to CD4^+^ T cells and induction of IFN-γ production, eliciting a protective Th1 response against herpes simplex virus 2 (HSV-2) (210). Though not involved in the response against viral HSV-2 infection, exposure to the BV-associated bacterium *Prevotella timonensis* renders LCs more susceptible to uptake of HIV-1 viral particles (224). Individuals with low *Lactobacillus* abundance and higher microbial diversity have increased proinflammatory cytokine abundance (216), and though the abundance of DCs, monocytes, and macrophages was not significantly different between individuals with high vaginal diversity and those with a *Lactobacillus*-dominant vaginal microbiota, there are significant differences in the transcriptomes across these APC populations (216). One potential source of this cellular signal is postulated to be cell envelope-associated, as the target genes identified in these transcriptomic analyses were closely related to responses of APC stimulated with lipopolysaccharide; however, many organisms associated with high diversity do not produce LPS, warranting more studies in this area (216).

### The role of secreted factors in reproductive tract immune surveillance

In addition to the cellular immune response, secreted factors such as antimicrobial peptides, chemokines, and cytokines, among others, help to facilitate the host defense. One notable secreted factor produced primarily by the vaginal epithelium is the alarmin, IL-33. Production of IL-33 is normally suppressed by the vaginal flora; however, in conditions of dysbiosis, the epithelium secretes IL-33 as a danger signal, inhibiting IFN-γ production, increasing host susceptibility to HSV-2 (225). Another secreted factor that is uniquely expressed in the reproductive tract and is critical for protection against pathogen invasion is interferon epsilon (IFN-ε) (226, 227). Expression of IFN-ε is constitutively expressed in the vaginal and endocervical epithelium but hormonally regulated at the endometrium (227, 228), where expression is inversely correlated with expression of the progesterone receptor, leading to lower levels of IFN-ε in the follicular phase and higher levels in the luteal phase (227). IFN-ε has been shown to have a protective role against *Chlamydia* infection and viral infections HIV, Zika virus, and HSV-2 (226, 228–233). Additionally, increased IL-17 has been observed in cervicovaginal lavages from individuals with active STIs compared to uninfected individuals and was found to be independent of the numbers of Th17 cells present (234). It is important to note, however, that IL-17 can be produced by myriads of cell types including neutrophils, mucosal-associated invariant T cells (MAITs), macrophages, etc. Cationic antimicrobial peptides (CAMPs) are also differentially expressed in the presence of BV-associated microbes as compared to a *Lactobacillus*-dominant microbiota (235), and a recent meta-analysis of vaginal metatranscriptomes described an increased profile of genes involved in CAMP resistance in samples collected from BV-associated microbiomes compared to samples collected from non-BV microbiomes (217). In the vaginal tract, both microbes and host cells are capable of secreting surfactant molecules that modulate the environment, ultimately impacting microbial colonization and the host response to infection. Host surfactants opsonize fungal cells through interactions with carbohydrate moieties, signaling them for phagocytosis (236–240). Recently, bacterial surfactants produced by *L. crispatus* were shown to reduce *Candida* species adherence to epithelial cells and inhibit *Chlamydia trachomatis* infection of host cells (241, 242). Similarly, surfactants produced by *L. gasseri* prevented biofilm formation of methicillin-resistant *S. aureus* (243), suggesting that surfactants may play a direct role in establishing and shaping the microbial communities of the vaginal tract.

### Nutritional immunity: immune modulation of the vaginal environment

Nutritional immunity refers to the host defense mechanism involving antimicrobial peptides that sequester available nutrient metal ions in an attempt to suppress growth of invading pathogens (244). Effector proteins such as the S100 family proteins, lipocalin, and lactoferrin can chelate metals and contribute to host defense (18, 245–248). Calprotectin is a tetraheterodimer of S100A8 and S100A9 that can sequester zinc, manganese, and iron (249–252). S100A7, or psoriasin, has affinity for zinc, and S100A12, or calgranulin C, has affinity for both zinc and copper (253–256). Lipocalin, also known as lipocalin-2 or neutrophil-associated gelatinase lipocalin, and lactoferrin are iron-chelating molecules that bind siderophores and ferric iron, respectively (257–261). Many of the nutritional immune effector molecules are expressed by host keratinocytes, epithelial cells, and innate immune cells, primarily neutrophils, and are found abundantly during periods of inflammation (249, 258, 262–265). Concentrations of lipocalin and calprotectin have been detected at higher abundance in communities where *L. iners* is dominant compared to *L. crispatus* dominant communities (44). In contrast, one study found that vaginal lipocalin was more abundant in a healthy control population compared to patients with BV, which was proposed as possibly due to the immunosuppressive potential of BV-associated microbes (130, 266). Vaginal lactoferrin levels were originally observed to increase just after menstruation and have been associated with increased vaginal microbial diversity (17, 18); however, new evidence suggests that in some individuals, the windows of time during and immediately after menstruation are inherently associated with increased microbial diversity, which could serve as a link between these two findings (6, 15). Collectively, these molecules, among others, are capable of restricting bioavailable nutrient metals and contribute to reducing invasion or expansion of pathogenic microbes including *C. albicans, Neisseria gonorrhea*and the opportunistic pathogen, *S. agalactiae* (130, 188, 258, 263, 267–270). In addition to their roles in nutritional immunity, the S100 family of proteins also function as alarmins or damage-associated molecular patterns and are capable of activation and amplification of the innate immune response (247, 271–273). Calprotectin has been shown to promote inflammation through the antagonism of toll-like receptor 4, and many of the S100 family proteins bind to the receptor for advanced glycation end products, leading to the activation of NFкB (263, 272, 273). These data indicate that the S100 proteins not only serve as metal chelators but may functionally promote inflammation, which is an understudied area of investigation in the context of the vaginal environment and has large implications for vaginal health. Few studies have sought to determine the availability of metals in this environment (17, 274, 275), but large gaps in knowledge still exist. Key questions remaining include understanding the impact of diet on availability, the impact of metal intake on host susceptibility or resilience to infection, the role in shaping community assembly, and understanding how microbes in complex communities access nutrient metals when the host is actively withholding.

## CONCLUSIONS AND FUTURE PERSPECTIVES

The vaginal landscape is a dynamic, intricately connected, ecosystem shaped by interactions between microbial communities and the host that fluctuate across changes in hormone signaling, barrier integrity, and nutrient availability. Here, we present this environment through an ecosystems perspective, with the goal of conveying the complexities and highlighting the gaps in knowledge existing in this system. Initial observations in the vaginal microbiome field were based on correlative data, but studies are now beginning to push toward understanding community interactions and consequences of change. Some of the large outstanding areas of investigation in the field include (i) understanding the drivers of community change: is the host modulating nutrient availability to select for a given microbial community, or does microbial metabolism drive environmental change?; (ii) clarifying the definition of “vaginal health”: is this guided by the microbes present, or should it be an individualized answer, where “health” is dictated by the absence of clinical symptoms? In this vein, continuing to assess the efficacy of BV diagnosis and treatment by Amsel and Nugent scoring rather than clinical symptoms; (iii) defining the idea and implications of “community dominance”: some studies present microbial dominance as >50% of a community (40), while others define dominance as >30% of a community (121); does one community convey a higher degree of protection than another?; (iv) understanding the contributions of lower abundance species: the vaginal tract is often portrayed as a monoculture of *Lactobacillus* but is much more diverse than this; if *Lactobacillus* is the critical player in these communities, why then do low abundance organisms persist in this inhospitable environment?; (v) determining the contribution of eukaryotic microorganisms, archaea, eukaryotic viruses, and bacteriophage, as these are widely understudied in the vaginal tract (276).

Innovative *in vitro* “on-a-chip” models and increases in longitudinal *in vivo* and clinical studies will continue providing insight into temporal fluctuation in the vaginal tract and will reduce the “snapshot in time” approach to understanding microbial community dynamics. Areas where our field can continue to grow include studies centered on diverse populations, including individuals outside of reproductive age *cis* women, such as postpartum, perimenopausal, menopausal, postmenopausal, and individuals undergoing gender-affirming care including transgender men and transgender women having undergone vaginoplasty. These populations are at increased likelihood for microbiota shifts away from *Lactobacillus* dominance but have been largely excluded from vaginal microbiome studies (111, 117, 218, 277–280). Additional improvements can be made in the use of neutral language when describing vaginal health, as use of words like “dysbiotic” or “non-optimal” has negative connotations which could perpetuate the already rampant stigma associated with vaginal medicine (281–284). An added caveat of this terminology is that individuals described as having “dysbiotic”, “non-optimal”, or non-*Lactobacillus*-dominant vaginal microbiota most prevalently include individuals identifying as Black, African American, Hispanic, or Latin. It is important to consider that this is likely not due to true biological difference but rather effects of social determinants of health including access to food and healthcare, socioeconomics, etc. (285). Studies centering on diversity in ethnic groups, cultural practice, impacts of systemic racism, influence of non-sex hormone signaling, and medical history, including chronic illness and sexual practice, will all provide additional insights into the vaginal microenvironment and their impacts on the resident microbes. This field has experienced impressive growth in recent years, and as we move forward, we should continue to push the bounds toward a more holistic, inclusive understanding of the vaginal environment and microbial community functions to advance our knowledge of vaginal health and improve care for all individuals.

## AFTER EPUB

*[This article was published on 11 August 2025 missing the author biographies. The author biographies were added in the current version, posted on 19 August 2025.]*
